# You pray to your God: A qualitative analysis of challenges in the provision of safe, timely, and affordable surgical care in Uganda

**DOI:** 10.1371/journal.pone.0195986

**Published:** 2018-04-17

**Authors:** Katherine Albutt, Rachel R. Yorlets, Maria Punchak, Peter Kayima, Didacus B. Namanya, Geoffrey A. Anderson, Mark G. Shrime

**Affiliations:** 1 Program in Global Surgery and Social Change (PGSSC), Harvard Medical School, Boston, Massachusetts, United States of America; 2 Department of Surgery, Massachusetts General Hospital (MGH), Boston, Massachusetts, United States of America; 3 David Geffen School of Medicine at UCLA, Los Angeles, California, United States of America; 4 Mbarara University of Science and Technology (MUST), Mbarara, Uganda; 5 Ministry of Health (MOH), Kampala, Uganda; 6 Uganda Martyrs University (UMU), Nkozi, Uganda; 7 Department of Otolaryngology, Massachusetts Eye and Ear Infirmary (MEEI), Boston, Massachusetts, United States of America; University of California Los Angeles, UNITED STATES

## Abstract

**Background:**

Five billion people lack access to safe, affordable, and timely surgical and anesthesia care. Significant challenges remain in the provision of surgical care in low-resource settings. Uganda is no exception.

**Methods:**

From September to November 2016, we conducted a mixed-methods countrywide surgical capacity assessment at 17 randomly selected public hospitals in Uganda. Researchers conducted 35 semi-structured interviews with key stakeholders to understand factors related to the provision of surgical care. The framework approach was used for thematic and explanatory data analysis.

**Results:**

The Ugandan public health care sector continues to face significant challenges in the provision of safe, timely, and affordable surgical care. These challenges can be broadly grouped into preparedness and policy, service delivery, and the financial burden of surgical care. Hospital staff reported challenges including: (1) significant delays in accessing surgical care, compounded by a malfunctioning referral system; (2) critical workforce shortages; (3) operative capacity that is limited by inadequate infrastructure and overwhelmed by emergency and obstetric volume; (4) supply chain difficulties pertaining to provision of essential medications, equipment, supplies, and blood; (5) significant, variable, and sometimes catastrophic expenditures for surgical patients and their families; and (6) a lack of surgery-specific policies and priorities. Despite these challenges, innovative strategies are being used in the public to provide surgical care to those most in need.

**Conclusion:**

Barriers to the provision of surgical care are cross-cutting and involve constraints in infrastructure, service delivery, workforce, and financing. Understanding current strengths and shortfalls of Uganda’s surgical system is a critical first step in developing effective, targeted policy and programming that will build and strengthen its surgical capacity.

## Background

Access to safe surgery is critical to health, welfare, and economic development. Annually, an estimated 17 million lives are lost from conditions requiring surgical care and at least 77.2 million disability-adjusted life-years could be averted through provision of basic surgical services [1]. Until recently, surgery has been largely omitted from the global public health discourse. While many have thought of surgery as too expensive and too resource-intense for low and middle- income countries (LMICs), recent data have shown that investment in surgical and anesthesia services is not only affordable and cost-effective, but that it also promotes economic growth [1]. Despite this knowledge, multiple barriers continue to prevent the provision of safe, affordable, and timely surgery to those who need it.

The burden of surgical disease disproportionately impacts low-resource settings, and Uganda is no exception. With a population of approximately 37 million people, Uganda ranked 163rd out of the 188 countries surveyed for the 2016 Human Development Index [2]. Since 1994, the country has had a decentralized health care system based on a hierarchy of increasing specialization. As of November 2016, there were 43 general hospitals (GHs), 14 regional referral hospitals (RRHs), and 2 national referral hospitals (NRHs), one of which is exclusively for mental health, in the public health sector [3]. The majority of Ugandans who fall sick (51%) first seek healthcare from a public health facility, followed by private and private not-for-profit health facilities [3]. Access to surgical services in Uganda is severely limited, largely by constraints in human resources, infrastructure, and supplies [4]. A 2016 cross-sectional nationwide household-based survey estimated that 3,680,000 Ugandans have unmet surgical need, in which 1,380,000 require surgical treatment and 2,300,000 require at least a surgical consultation [5]. To understand the capacity of the healthcare system to deliver safe, timely, and affordable surgical care in Uganda, we partnered with the Ministry of Health to conduct a countrywide surgical capacity assessment.

## Methods

The qualitative data presented in this article were derived from a larger mixed-methods nationwide study conducted in Uganda from September to November, 2016. Qualitative analysis is presented in this manuscript; quantitative results will be published elsewhere [6].

### Surgical Assessment Tool

A Surgical Assessment Tool (SAT), jointly developed by the Program in Global Surgery and Social Change at the Harvard Medical School (PGSSC) and the World Health Organization, was employed at each hospital between September and November 2016. The quantitative portion of the assessment involved a combination of hospital walk-throughs, retrospective reviews of operative logbooks, and interviews with hospital directors and clinicians. The qualitative portion of the assessment was comprised of in-person semi-structured interviews of key stakeholders to understand the factors related to the provision of safe, timely, affordable surgical care. The qualitative interview tool can be found in S1 Fig. Open-ended questions were asked regarding infrastructure, service delivery, workforce, information management, and financing. Guiding questions included examples of probes to be used by interviewers as follow up to specific questions.

### Sampling frame

**Fig 1:**
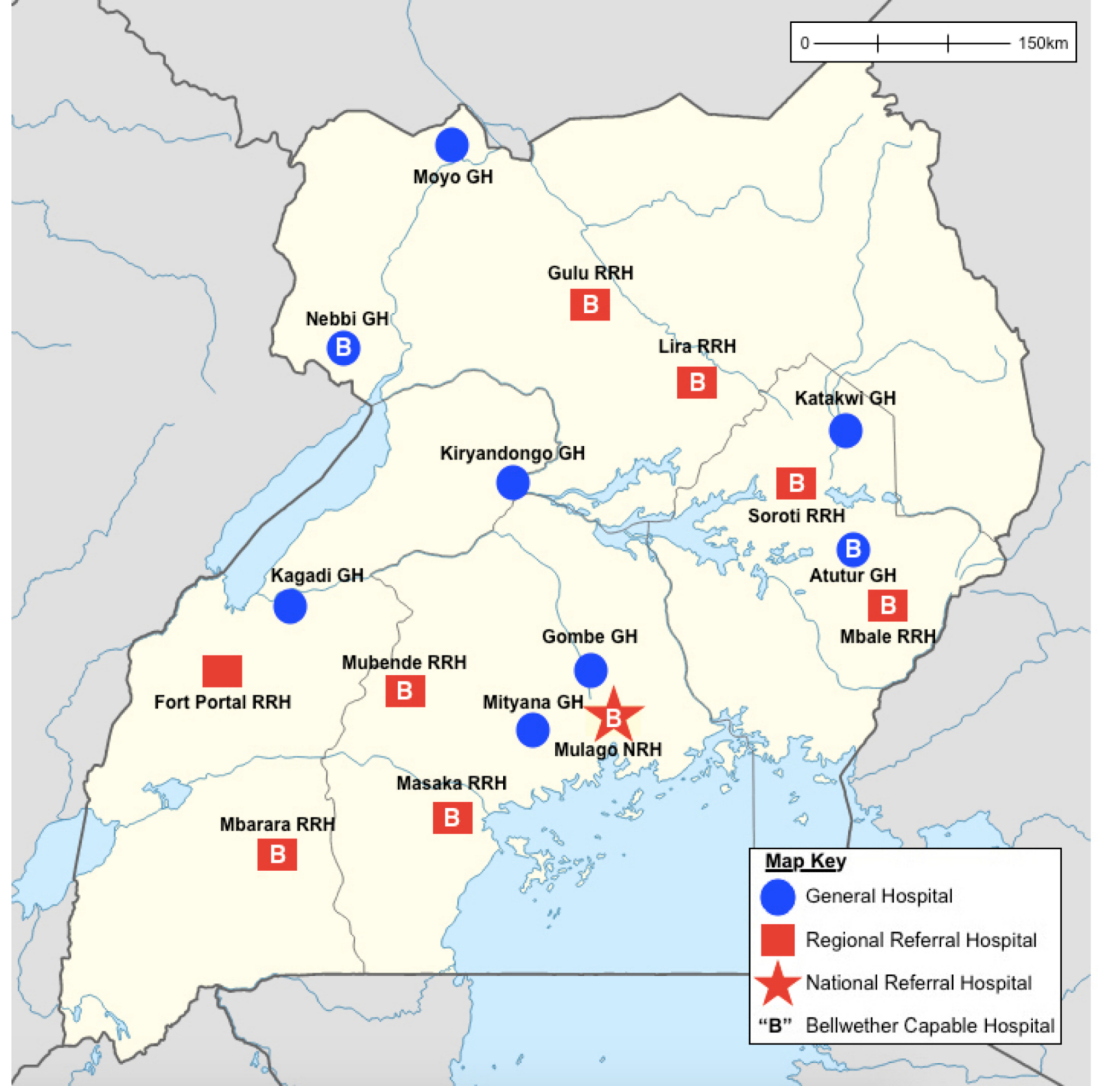
Map of selected facilities.

Data were collected at 17 randomly-selected government hospitals in Uganda. Stratified random sampling was used to select 2 facilities at the GH level and 2 facilities at the RRH level per region, plus the national referral hospital, to ensure a geographically representative sample (Fig 1). Data collection was conducted by a team composed of four researchers, including a global surgery fellow and global surgery research associate from the PGSSC, representative from the Ministry of Health, and a Ugandan surgeon. Site visits were conducted at one facility per day and took an average of 3–6 hours.

### Qualitative study procedures

Eligible participants included hospital directors and administrators, surgeons, obstetricians, and principal nursing officers. During each hospital site visit, all eligible and available hospital staff were recruited to participate in a qualitative interview. Verbal informed consent was obtained prior to enrollment. The most senior-level provider, or their designee, was interviewed within each cadre. Participants were excluded from this group if they were unavailable to participate. Individual in-depth interviews were conducted in English (an official language in Uganda) in a private setting and responses were recorded with a recording device; interview duration ranged from 30 to 60 minutes.

### Coding and analysis

Each interview was transcribed verbatim by a member of the study team. Interview transcripts were subsequently cross-checked for accuracy and completeness by another member of the study team. Identifying information was removed to preserve anonymity and confidentiality of participants. Electronic transcripts were uploaded to the qualitative data analysis software NVivo (QSR International Nvivo 11) [7]. Two authors (KA and RY) reviewed the first five transcripts to identify salient topics and themes and to develop a coding manual. This manual was then used by the primary analyst (RY) to code all subsequent transcripts with independent validation conducted by a second analyst (KA). RY has a public health background and has worked in global surgery research for a number of years, KA is a surgical resident and global surgery research fellow who has worked extensively in sub-Saharan Africa. The data were subsequently organized into key conceptual themes and thematic content analysis was used as the analytic approach. Thematic saturation was achieved. Coding inter-rater reliability, measured with a pooled Cohen’s kappa, was 0.82 [8].

### Ethical considerations

This study was deemed exempt from review by the Institutional Review Board (IRB) at Boston Children’s Hospital and approved by the IRBs at Mbarara University of Science and Technology and the Uganda National Council for Science and Technology.

## Results

### Demographics of participants

A total of 35 interviews were completed with participants at 17 hospitals. Interviews were completed with 11 hospital directors or medical superintendents, nine physicians (surgeons, anesthesiologists, or obstetrician/gynecologists), eight principal/senior nursing officers, and four other designees (two anesthetic officers, a hospital administrator, and a nurse). For logistical reasons, three of these were group interviews that involved hospital administrators, physicians, nurses, and others. Of these interviews, 18 were performed at GHs, 16 at RRHs, and one at the NRH. Participants were asked questions semi-structured and open-ended questions regarding infrastructure, service delivery, surgical workforce, information management, and financing of surgical care.

### Patient population

Hospitals’ catchment populations were described as large, far-reaching, and serving up to several million people. Significant difficulties accessing timely surgical care were a recurrent theme across interviews. Many participants noted that patients travel long distances to reach health facilities, often coming from outside of the designated district or regional catchment area, including patients from refugee camps, other regions of Uganda, and even its neighboring nations. Hospital staff noted that large periods of time often elapse between symptom onset and the receipt of appropriate surgical care. Low health literacy and distrust of the healthcare system further contribute to delays. Many of the communities are poor, and, as one participant emphasized, *“…poverty usually goes with high disease burden”* (Participant 31). One participant identified boda bodas (motorcycles) as the *“number one killer”* and most maintained that trauma and emergencies overwhelm the surgical caseload.

### Infrastructure

Many health care workers reported that the facilities that provide surgical care were built decades ago and were designed to accommodate a much smaller catchment population. Consequently, theater and ward capacity are limited. Most clinicians and administrators reported that their hospital census exceeds bed capacity, and some patients end up being relegated to the floor: *“Most of the time there are more patients than beds*. *Floor cases are everywhere”* (Participant 16). Similarly, operating theatres are shared by many specialties, and planned procedures are often delayed for emergent ones. Many participants reported that there is no recovery room, monitored beds are scarce, and patients often recover in the hallway. Many voiced the need for construction to update and expand their facilities, but those with renovations in progress explained that ongoing construction rendered wards and theatres unusable for significant lengths of time.

Most participants reported being connected to the national grid, but all described their electricity supply as erratic and unpredictable. Outages are not infrequent, can be lengthy (lasting upwards of one to two weeks), and may result from service interruptions or lack of payment. Standby generators are sometimes broken and often not automatic. In some facilities, solar panels provide a backup source of energy, usually limited to lighting. While some estimated that water is more reliable than electricity, the supply is also erratic. Some hospitals rely on underground water, but this is not accessible when the power is out or the pump is non-functional, sometimes for weeks at a time. In the dry season, the supply is further constrained, forcing facilities to resort to bringing in jerry cans of water.

### Service delivery—Access to supplies, medication, oxygen, blood, and equipment

All participants described critical supply shortages and stock-outs, exacerbated by ineffective coordination between hospitals and National Medical Stores (NMS), the organization mandated to procure, store and distribute essential medicines and supplies to all public health facilities [9]. Supply is often variable, infrequent or poorly timed, and inadequate to meet high patient volumes. In almost all circumstances, demand far exceeds supply, leading to critical shortages of certain consumables, medications, and equipment.

Certain items are particularly vulnerable to stock-outs, especially urethral catheters, nasogastric tubes, intravenous fluid, gloves, and sutures. Clinicians reported that these critical supply shortages may cause delays or cost patients their lives, as you *“cannot risk going in without having certain supplies available”* (Participant 25). Speaking specifically regarding the availability of suture, one physician stated:

*Suture is problematic. Sometimes what you want is not there and you must improvise. Sometimes we send the attendants to source sutures from various places… Actually, many times you delay the surgery because you don’t have [suture]*.

(Participant 4)

Numerous participants described a mismatch between what they request and receive from NMS. For example, hospitals receive more sterile gloves than examination gloves, and when the latter runs out, clinicians use sterile gloves for non-sterile tasks, leading to severe shortages of the former. Speaking of the supply chain, one participant said:

*If the supplies have just been delivered it is fine… then we are sometimes completely down. And even then, the supplies from the National Medical Stores are extremely variable. Sometimes they don’t supply everything at once, sometimes they do. So, it can be really variable. Duration of delivery depends on what they have supplied. Sometimes they will miss out on a requested item completely. It is really hard to predict. If they supplied everything requested perhaps it would be easier to know*.

(Participant 19)

Meanwhile, facilities with few sets of surgical instruments are forced to stop mid-day to sterilize sets before continuing with cases:

*The biggest challenge I have in running this place is instruments. All forms of instruments. Because they are really, really very old. We try to use them because there is nothing else. They are also very few. I will tell you there are only two caesarean section sets and yet when the place is busy you are doing between five and 10 caesarean sections a day*.

(Participant 1)

Participants also described the delays and inconveniences caused by the need to borrow supplies, refer patients because of supply chain constraints, or ask patients’ attendants (family/caregivers who assume a significant portion of the care-taking burden while patients are hospitalized) to purchase materials. Despite these supply-side limitations, providers note that they rapidly adapt to this context. Innovations include but are not limited to using foley catheters as chest tubes, utilizing water filled plastic bottles to simulate a water-seal chest drainage system, and employing various suture conserving techniques, amongst others.

Participants also described constraining shortages and stock-outs of medications critical to surgical care. Several reported that antibiotics are the first drugs to run out of stock and, as a result, patients are often asked to buy them. In many situations, hospital staff reported that broad-spectrum antibiotics may be indicated, but they can only prescribe that which is accessible, *“The choice will depend on what is available*, *not on what is recommended”* (Participant 29). Participants also described a variable supply of anesthesia drugs, explaining that this is again reflective of an inconsistent supply chain. One anesthesia clinician described the situation as particularly dire *“We have to use whatever materials are around*. *That is how we are working*. *We are just stumbling*. *You pray to your god before you give anesthesia”* (Participant 13). Supplies are also frequently unavailable for spinal anesthesia, forcing hospital staff to borrow from other hospitals, exceed the hospital budget for purchases, and ask patients to purchase.

Participants described a range of oxygen availability for their hospitals, from no oxygen supply to a fairly regular supply that enables them to share with nearby hospitals. Supply can be erratic and hospitals may have oxygen for part, but not all, of each week, and may be forced to rely on cylinders from far away. One participant stated:

*Right now, we do not have oxygen. But we have been in touch with NMS who have been telling us, ‘We are going to supply, we are going to supply.’ For a hospital like this one to not have oxygen, you are as good as useless. And eventually people begin blaming us*.

(Participant 12)

Hospitals without oxygen cylinders rely on oxygen concentrators, which only work when electricity is available. Inconsistent oxygen supply limits ability to provide care and is the most frequent reason for case cancellation in many locations. As one participant described, *“I know that I used to cancel the [surgical] lists because of lack of oxygen*. *And now oxygen is here*, *at least we do not cancel very frequently because there is no oxygen”* (Participant 24). The supply of oxygen has been so variable and troubling that there are plans in place to attempt to construct oxygen plants at all RRHs (currently only one RRH has an oxygen plant).

Surgical care is also delayed when blood is needed. Patients’ access to blood depends on the availability, the location of the nearest regional blood bank, and whether the blood has been picked up by the hospital. However, even when a hospital staff member is sent to the blood bank, there is no guarantee that blood will be available. Participants explained that many people in the community are reluctant to donate, and blood banks rely heavily on students as donors with blood shortages increasing during school holidays. There is limited availability of less common blood types and blood is sometimes discarded after screening and due to the high prevalence of HIV and Hepatitis B. In cases where the blood supply is lacking, the indicated operation is frequently not performed and patients are referred to another facility, further exacerbating delays in care.

Provision of surgical care is also limited by equipment that is unavailable, non-functional, and outdated. Some pieces of equipment are completely unavailable (e.g., computed tomography scanners, incubators) and many others do not work, work only intermittently, or are rendered non-functional by electricity outages or missing materials (e.g., an anesthesia machine without a monitor, an ultrasound without gel, lighting equipment without bulbs). Outdated equipment is particularly susceptible to frequent breakdowns, making equipment maintenance an ongoing challenge. Despite the recurrent need for equipment repair, maintenance is regionalized, and engineers only visit the hospitals infrequently for short periods of time. Due to excessive volume, limited human resources, and high costs, engineers sometimes do not complete necessary repairs before leaving. In some cases, there is no service contract for equipment. To work around these challenges, clinicians and administrators purchase and use their own equipment, rely on donations from visiting surgical camps, take broken equipment directly to joint medical stores, ask biomedical engineers from other collaborations to work on machines, or seek private and sometimes costly technical assistance.

### The surgical workforce

Participants across facilities consistently reported a workforce shortage, spanning from specialists to support staff. Frequent references to urban/rural physician maldistribution, lack of specialists, and resultant understaffing of facilities were made. Health care worker morale suffers, as a result, with many practitioners overworked and frustrated by the inadequacies of the system. Numerous facilities function with a fraction of the needed specialists and senior physicians, while some specialty services (e.g., pediatrics, surgery, medicine) are not provided at all. Medical officers (non-specialist physicians who have completed an internship) shoulder a large portion of the surgical workload. One participant stated:

*According to structure, this place is supposed to have five specialists and six medical officers. Currently, there is one specialist and one medical officer. So that is how challenging the situation is. It is really not good. I am only enduring, I might also run away if the situation does not improve. And you know, the attrition rate is really very high. It is attraction and retention that are very hard and it all [comes] down to motivation. The human resources are lacking. People find it attractive to work in places where there is better equipment to use, other services are available, they are motivated, even the money is a factor*.

(Participant 1)

Nurses are similarly understaffed with nurse-to-patient ratio estimates ranging from 1:10 to 1:60. Additionally, several participants remarked that the government is phasing out nursing assistants and nursing aides.

In many cases, the technical training of the existing workforce may be inadequate and insufficiently specialized. This is exacerbated by a lack of continuing education. Health care workers of all levels sometimes work alone (e.g., one nurse covers an entire ward overnight), and task-shift (e.g., medical officers perform cesarean sections and other operative procedures). Furthermore, many physicians take on administrative and leadership responsibilities. Numerous clinicians explained that they work without any clinical back-up and may not be able to refer their patients because there is no health care worker at the referral facility. Extreme staff shortages may not be temporary, *“For the last two years*, *I have been the only surgeon here*. *And I have to do so many other things also”* (Participant 1). Working without back-up can increase risk, as it makes it difficult to implement quality measures (e.g., WHO Safe Surgery Checklist) and increases risks for patients (e.g., junior surgeons pressured to operate at night when a consultant is unavailable).

### The referral system

Many of the aforementioned problems are exacerbated by a disorganized referral system. Despite health care theoretically being decentralized and based on a hierarchy of increasing specialization, hospital staff report that the system does not work and patients do not follow this system as it was designed. *“Most of the people come themselves*, *they are not referred”* (Participant 2). Such patterns of care seeking create an imbalance between supply and demand at the facility level:

*The ideal is that only cases needing [higher] level of care come [to the referral hospital] but that of course is not what happens. We see everything. We are doing the job of a health center right up to a national referral hospital. We struggle through*.

(Participant 19)

When one hospital does not have the resources or personnel necessary to treat a condition, the patient is frequently referred to the next level facility, despite there being no guarantee that the necessary resources or personnel will be available at that facility. Such referrals can occur for things as simple as sterile drapes and gowns:

*Some patients get referred out [due to unavailability of sterile] linen. For example, if you come with a ruptured uterus and we don’t have linen, we refer you. You imagine referring to [the nearest RRH], it is quite far. So, when she comes here from a very far village with a ruptured uterus and you don’t have linen, then [refer] to Fort Portal, sometimes they die in the ambulance. That’s the challenge*.

(Participant 8)

Others are referred due to lack of blood, suture, surgical blades, instruments, imaging capability, and personnel, amongst other constraints. These difficulties are further compounded by poor access to transportation and fuel, significant delays in the provision of care, and limited communication and coordination channels.

### Financing surgical care

Participants explained that these shortages of infrastructure, workforce, and supplies are underlined by a system that is financially constrained. The budget for hospitals is based on the antiquated estimates of the population of the district or region. Funds that are distributed each quarter are consumed quickly, making it difficult for facilities to save for critical purchases or to fund research. Additionally, surgery has not typically been considered a public health funding priority: *“Nobody is funding [surgery]*. *People think reproductive health*, *people think HIV*, *but nobody thinks surgery”* (Participant 5). One participant described the challenge of surgical care provision as one that ultimately comes down to financing:

*The challenge of surgery in Africa is funding. Infectious disease, a lot of money is spent on TB, on malaria, or on HIV, you see. Very little on trauma and yet we have also known that trauma is killing a lot of our patients*.

(Participant 24)

The government of Uganda abolished user fees in 2001, effectively rendering all care performed at government facilities free of charge. Despite this policy being in existence, the supply chain constraints described above often limit facilities to such an extent that costs for unavailable resources end up falling to the patients and their families: *“This is a government hospital so when they come*, *everything is supposed to be free*. *Unless those things are not there*, *that is when they will meet a cost*. *But if they are there*, *they get freely”* (Participant 2).

Another health care worker echoed these sentiments, stating:

*In case any supply is not there, as managers, what do we do? We throw the burden to the service seekers. They have to provide them. Because if the national supplier does not deliver any item, then we don’t have another option… So because of that, the whole weight is thrown back to [the patient]. But if the supplies are there, we don’t ask [the patient] to buy anything—not gloves, nothing*.

(Participant 16)

## Discussion

**Fig 2:**
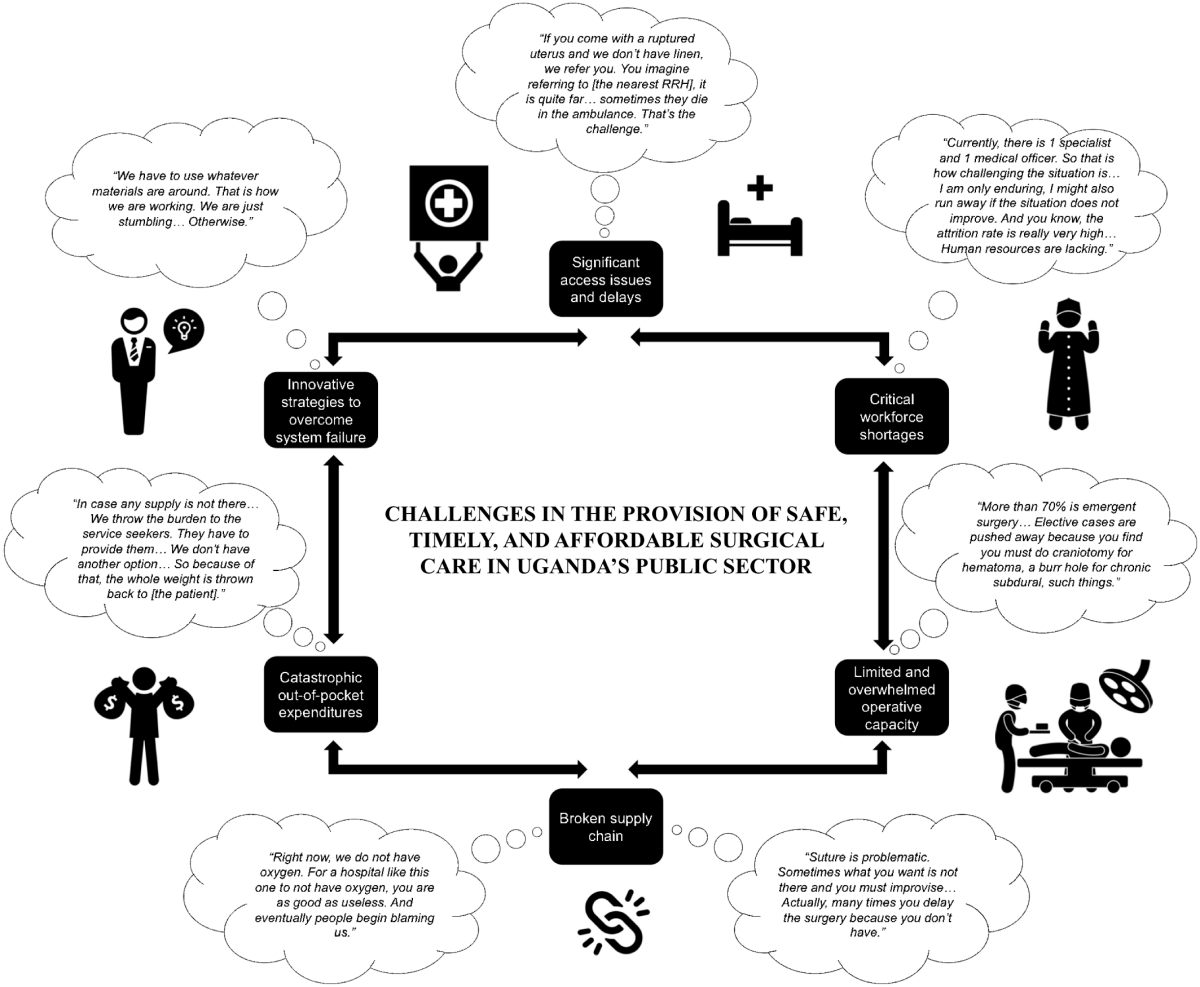
Challenges in the provision of surgical care.

This manuscript highlights the voices of health care workers on the front lines of surgical care delivery in Uganda’s public health care sector and highlights the tremendous challenges in providing safe, timely, and affordable surgical care. The barriers to the provision of surgical care are cross-cutting and involve constraints in infrastructure, service delivery, workforce, and financing. While some differences exist between facilities, the barriers to safe surgery and anesthesia are overall consistent and resource-driven. These challenges can be broadly grouped into six categories: (1) significant delays in accessing surgical care, compounded by a malfunctioning referral system; (2) critical workforce shortages; (3) operative capacity that is limited by inadequate infrastructure and overwhelmed by emergency and obstetric volume; (4) supply chain difficulties pertaining to provision of essential medications, equipment, supplies, and blood; (5) significant, variable, and sometimes catastrophic expenditures for surgical patients and their families; and (6) a lack of surgery-specific policies and priorities (Fig 2).

In Uganda, patients often experience delays in seeking, accessing, and receiving surgical care that severely impede reduction of surgical morbidity and mortality. Currently, an estimated 5 billion people worldwide are unable to reach surgical services when considering the four dimensions of access: timeliness, surgical capacity, safety, and affordability [1,10]. Ultimately, access is multi-faceted and involves geographic, temporal, financing, sociocultural, and political constraints. Not surprisingly, the access chasm is worse for those in LMICs, like Uganda, and those in the poorest wealth quintiles [1,11]. Clinicians and administrators cite lack of access to surgical care as one of their biggest obstacles. In nearby Zambia, upwards of 80% of the population lack access to trauma care, obstetric care, and care of common abdominal emergencies [12]. Across the continent, the best available models suggest that up to 95% of the population might lack adequate access to surgical care [10]. Unfortunately, the situation in Uganda is no different with less than 25% of the population being able to reach a facility capable of providing basic and essential surgical care within two hours [6]. The metric of two-hour access is one of six core surgical indicators identified by the *Lancet* Commission on Global Surgery (LCoGS) as illustrative of the strength of a surgical system. Without significant political and financial investment, Uganda is not likely to meet the 80% accessibility target set by LCoGS by 2030.

Access and availability of surgical services in Uganda can be conceptualized using the Three Delays Framework, as has been done in a 2016 multi-country qualitative study by Raykar et. al [13]. The delay in seeking care, the first delay, occurs when health-seeking behavior is limited by geography, finances, education, cultural beliefs, and/or a lack of confidence in available services [14]. Results from a breast cancer study in Uganda found that more than 77% of patients presented with stage III or IV disease, suggesting that patients do not seek and/or reach care in a timely fashion [15]. In Uganda, clinicians note that patients often present late and in critical condition.

The second delay, or the delay in reaching care, occurs when surgically-capable hospitals are scarce and may be miles and hours to days away with extremely limited transportation options [16]. For 2.2 billion people worldwide, access to an operating room is out of reach [17]. In fact, in India, populations living more than 50 kilometers from well-resourced district hospitals were four times as likely to die from acute abdominal conditions as compared to those living closer [18]. Surveyed clinicians and administrators in Uganda consistently noted that patients do not reach care in a timely fashion.

The third delay, the delay in receiving care, occurs when a facility cannot provide requisite treatment for a patient, prompting referral and exacerbating existing delays. Despite the recommendation that district-level hospitals should be able to basic and essential surgical procedures (including the bellwether procedures—laparotomy, cesarean section, and open fracture repair), clinicians and administrators noted that patients are often transferred because of inadequate capabilities at the district hospital level [1]. Furthermore, the referral system within the public health care sector is marred by communication and transportation difficulties, with no guarantee that the receiving facility can provide the requisite care.

If patients are ultimately able to reach care, they often find themselves at facilities facing critical workforce shortages. Hospital staff frequently referenced the Ugandan surgical workforce crisis, maldistribution, and resultant understaffing. On a more global scale, it is well established that LMICs are disproportionately affected by low surgical workforce density. Despite housing almost half of the world’s population, low- and lower-middle-income countries only have 20% of the health workforce (19% of all surgeons, 15% of anesthesiologists, and 29% of obstetricians) [19]. Within these countries, people living in rural areas, those with a low income, and those who are marginalized are the most affected by gross shortages and maldistribution of clinicians [18]. Deficits in education, training, and loss of clinicians to the private sector and other countries compound this issue [20]. The second core surgical indicator, specialist surgical workforce density, is born from the observation that higher provider density correlates with improved maternal survival, with particularly steep improvement towards improvements up to the threshold of 20 specialist providers per 100,000 population [1]. In Uganda, there are currently only 0.3 surgical specialist providers per 100,000 in the public health care sector [6]. Worldwide, an estimated 1.27 million providers need to be trained for all countries to reach a specialist surgical workforce density of 20 specialist physicians per 100,000 population by 2030 [21]. Innovative strategies to expand, maintain, and improve the existing surgical workforce are of paramount importance if surgical care delivery is to improve in Uganda.

Furthermore, inadequate infrastructure and high obstetric and emergency surgery volumes overwhelm already limited operative capacity according to hospital staff. When surgical care is not available in a timely manner, easily treatable conditions become diseases with high case fatality rates [1]. Obstructed labor may result in maternal and neonatal mortality, hernias become incarcerated, and broken bones lead to life-long disability. Elective cases may wait days, weeks, or even months to years for available operating room space [14]. Tertiary centers are overcrowded with overflow from poorly-functioning district hospitals and thus lose their ability to perform more complex elective surgery [22].

The overwhelming emergency, obstetric, and referral case volume is compounded by the infrastructural deficits that often trouble hospitals in LMICs. A survey of almost 800 facilities in low-income countries noted that 31% did not have reliable electricity, 22% did not have running water, and 24% did not have oxygen [23]. Furthermore, available evidence suggests that it is not only the availability of resources, but also variability in their supply that leads to system breakdowns and inefficiency [24]. A minimum of 143 million additional surgical procedures each year are necessary to save lives and prevent morbidity with the need greatest in the poorest regions of the world, including sub-Saharan Africa [1,25]. The target of 5,000 procedures per 100,000 population, another core surgical indicator, is the proposed minimum recommended threshold to support desirable health outcomes [26]. At the time of this assessment, the surgical density in Uganda was 144.5 cases per 100,000 people per year, suggesting that the surgical case volume is well below desirable levels [6].

In the Ugandan public health care sector, the aforementioned difficulties are exacerbated by supply chain difficulties and stock-outs of medications, equipment, supplies, and blood. Often the result of insufficient funding and poor logistical management, the absence of a stable surgical supply chain impedes the provision of surgical and anesthesia care delivery [27–30]. Uganda is one of many LMICs where surgical facilities run at or close to the minimum inventory [31]. Quality, safe, and affordable consumables and equipment are often unavailable, unreliable, and insufficient to meet demand. Significant investments in information management, communication, and supply chain efficiency could help avert critical shortfalls and dramatically improve surgical system functioning. Furthermore, health care workers in Uganda have become accustomed to innovating novel techniques and uses of equipment in the setting of resource limitations. Such techniques can and should be documented, assessed, and made accessible to providers across settings struggling with similar supply-side shortcomings.

While health care at government facilities is legally mandated to be provided free of charge in Uganda’s public health care sector, the reality is that patients and their families experience significant, variable, and sometimes catastrophic expenses due to seeking surgical care. This situation is not unique to Uganda, with out-of-pocket payments representing one of the most predominant forms of healthcare financing worldwide [32,33]. Every year, an estimated 81.3 million people face financial catastrophe as a result of surgical care and associated non-medical costs. Not surprisingly, according to hospital staff, this burden is disproportionately shouldered by the poor [34]. Increasingly, global health and multilateral organizations have supported prioritization of financial risk protection, a goal reflected in the LCoGS indicators of protection from catastrophic and impoverishing expenditure [35,36].

Lastly, hospital staff across settings noted seemingly outdated and unresponsive national health policies that do not reflect current realities. As summarized in a recent multi-national qualitative study by Raykar et al.: “Providers consistently voiced their frustration that, in addition to working within an underfunded health system, they are governed by policies that do not align with the realities of low-resource care provision” [37]. Budgetary allocations, staffing, and disbursement of supplies are based on antiquated population estimates and often do not reflect the care-seeking behavior of the population. Large mismatches between supply and demand often exist (i.e. there are often spinal needles without spinal anesthesia or sterile gloves without examination gloves) and much equipment is donated, foreign, prone to breaking, and with little yet complicated serviceability (assessments show that almost 40% of donated equipment is out of service) [38–40]. With little attention paid to context or reality on the ground, it is not surprising that existing policies and procedures do not maximally support efficient surgical care delivery.

### Limitations

The qualitative data presented here are derived from a larger mixed-methods study conducted at 17 public hospitals around Uganda. Responses were audio-recorded, and errors and biases may have occurred in transcription, although every transcription was cross-checked by a second research team member. Responses may have been influenced by the interviewers’ own biases and perceptions, and the presence of the interviewer may have in turn impacted the response. Furthermore, due to logistical limitations several interviews occurred in a group setting which may have limited participant’s ability to be candid. Recall bias among study participants may have also been an issue. Efforts were made, however, to utilize interviewers who were trained in qualitative research methods, comfortable with the sensitivities of the subject matter and cultural norms, and knowledgeable in how to probe for more accurate recall. Finally, bias may have been introduced during data analysis, although this was minimized by not including the primary analyst in the interview or transcription process, and through dual codebook development and independent coding.

## Conclusion

Understanding current strengths and shortfalls of Uganda’s surgical system is a critical first step in developing effective, targeted policy and programming that will build and strengthen its surgical capacity. Augmenting the capacity of the public health care sector, as the provider of care for the majority as well as the most impoverished, is essential to improve surgical care in Uganda. The voices of front line health care workers, often unheard in higher-level policy and programming discussions, add valuable granular detail to the challenges and complexities of providing surgical care in LMICs. There is a vital role for qualitative data while assessing surgical capacity and designing policy and programming to improve access to safe, timely, and affordable surgical care. Unfortunately, surgery has historically been a neglected global health discipline in terms of programming, policy, and funding, both in Uganda and elsewhere. If the surgical access chasm is to lessen, surgery must be proactively addressed and acknowledged as a critical component of universal health care. One potential avenue for investing in surgery on the national level is through the creation of a national surgical, obstetric, and anesthesia plan: a country-led plan for scaling up surgical and anesthesia care as part of health system strengthening. Further research is warranted to better understand the impact of surgical capacity building interventions as they are undertaken in LMICs.

## Supporting information

S1 Fig Semi-structured interview tool. (PDF)
